# Long-term effects of Youth Mental Health First Aid training: randomized controlled trial with 3-year follow-up

**DOI:** 10.1186/s12888-020-02860-1

**Published:** 2020-10-06

**Authors:** Amy J. Morgan, Julie-Anne A. Fischer, Laura M. Hart, Claire M. Kelly, Betty A. Kitchener, Nicola J. Reavley, Marie B. H. Yap, Anthony F. Jorm

**Affiliations:** 1 Centre for Mental Health, Melbourne School of Population and Global Health, University of Melbourne, 207 Bouverie Street, Melbourne, Victoria 3010 Australia; 2 School of Psychology and Public Health, La Trobe University, Melbourne, Australia; 3 Mental Health First Aid Australia, Parkville, Australia; 4 School of Psychology, Faculty of Health, Deakin University, Burwood, Australia; 5 School of Psychological Sciences, Turner Institute for Brain and Mental Health, Monash University, Clayton, Australia

**Keywords:** Mental health first aid, Social support, Help-seeking behavior, Mental disorders, Adolescent, Parent

## Abstract

**Background:**

Mental Health First Aid (MHFA) training teaches community members how to provide initial support to someone with a mental health problem. Key gaps in the evidence base supporting the training are the longevity of effects beyond 6 months, effects on mental health first aid behavior, and the impact of support on the recipient of aid. This study aimed to evaluate the effect of the Youth MHFA course 3 years after training.

**Methods:**

384 Australian parents of an adolescent aged 12–15 were randomized to receive either the 14-h Youth MHFA course or the 15-h Australian Red Cross Provide First Aid course. This paper reports outcomes at baseline and 3 years later. Primary outcomes were cases of adolescent mental health problems, and parental support towards their adolescent if they developed a mental health problem, rated by the parent and adolescent. Secondary outcomes included parent knowledge about youth mental health problems, intentions and confidence in supporting a young person, stigmatizing attitudes, and help-seeking for mental health problems. Data were analyzed with mixed-effects models with group by measurement occasion interactions.

**Results:**

3-year follow-up data was obtained from 149 parents and 118 adolescents, who were aged 16.5 years on average. Between baseline and 3-year follow-up, there was a non-significant reduction in adolescent cases of mental health problems relative to the control group (odds ratios (OR) 0.16–0.17), a non-significant improvement in parental support reported by adolescents with a mental health problem (OR 2.80–4.31), and a non-significant improvement in the quality of support that parents reported providing to their adolescents with a mental health problem (d = 0.38). Secondary outcomes that showed significant improvements relative to the control group were parental knowledge about youth mental health problems (d = 0.31) and adolescent perceptions of general social support from their parents (d = 0.35).

**Conclusions:**

This paper reports on the longest follow-up of Mental Health First Aid training in a controlled trial. Three years after training, participants had maintained their improved knowledge about mental health problems. There were some indications of other positive effects, but the study was underpowered to clearly show benefits to mental health first aid skills and recipients of aid.

**Trial registration:**

ACTRN12612000390886, registered retrospectively 5/4/2012, https://www.anzctr.org.au/Trial/Registration/TrialReview.aspx?id=347502

## Background

Mental Health First Aid (MHFA) is a training program that teaches community members how to provide initial support to people with mental health problems. MHFA training was developed because members of the public often have poor mental health literacy, possess stigmatizing attitudes, and lack the confidence and knowledge of how to help a person with a mental health problem [1]. Yet friends and family are often the first people who can provide assistance and facilitate professional help. MHFA teaches people how to assist those who are developing a mental health problem, experiencing a worsening of an existing mental health problem or are in a mental health crisis. The course is analogous to physical first aid, where aid is given until professional help is received or the crisis resolves. There are core MHFA courses to assist specific population groups (e.g. adults, youth, older people), and specialized courses teaching how to help someone experiencing a specific crisis or situation, such as suicidal thoughts and behaviors [2]. MHFA training began in Australia in 2000 and has had a global impact, spreading to over 27 countries with more than 3 million people trained worldwide [3].

One factor in MHFA’s successful dissemination has been its commitment to evidence-based training and strong research focus [4]. Training courses are based on guidelines on how to provide mental health first aid that are developed using expert consensus studies [5]. Key elements of the guidelines are summarized in a 5-point MHFA Action Plan taught in the course. MHFA courses are also regularly revised to incorporate new research, such as updated guidelines on how to provide mental health first aid (e.g. [6]). There has also been a strong emphasis on rigorous evaluation of courses. There have been at least 18 controlled trials of MHFA courses, conducted by different investigators around the world [7]. These evaluations have demonstrated improved knowledge about mental health problems and treatments, reduced stigma, and improved confidence and intended support towards people with mental health problems or in crisis. A smaller number of trials have also shown increased help offered to someone with a mental health problem [8–10]. While these findings provide support for the program’s benefits, there are some key evidence gaps. Few studies have investigated outcomes more than 6 months after training, so the longevity of the above effects is unclear. Furthermore, recent reviews [7, 11, 12] have highlighted that there is weak evidence supporting improved mental health first aid behavior, and few studies have investigated the effects upon the *recipient* of mental health first aid, rather than trainees. These effects include perceived support from the first aider, mental health service use, and improvements in mental health.

The Training for Parents of Teenagers randomized controlled trial was designed to address key limitations in the evidence base of the MHFA program. The trial aimed to investigate the long-term effects of the training and its impact upon the recipient of aid. Parents of early adolescents were trained in Youth Mental Health First Aid, and effects upon their teenage children were investigated. The training was delivered during adolescence, a critical period when mental health problems often first develop and mental health first aid could therefore be offered [13]. The use of an active control condition (physical first aid training) meant that effects could be assessed several years after training, which is impossible to do with a waitlist control design. We have already reported outcomes from the trial at 1-year and 2-year follow-up [14], which showed improvements in mental health literacy in training recipients relative to the control. For example, increased parental knowledge about youth mental health problems (d = 0.43 at 1-year and d = 0.26 at 2-year), and increased confidence to help a young person (d = 0.26) and intentions to provide effective support (d = 0.22) at 1-year follow-up. However, effects on the recipient of aid were less clear. The number of adolescents with a mental health problem during follow-up was too small to provide certainty about the effects of the training. There were very small, non-significant differences in the quality of parental support, favoring the MHFA group (ds = 0.16–0.17). Additionally, although the proportion of adolescents with a mental health problem dropped in the MHFA group but remained similar in the control group, the study was underpowered to detect a difference.

This paper reports on 3-year follow-up outcomes from the Training for Parents of Teenagers study. This is the longest follow-up yet of a controlled trial of MHFA. Furthermore, given the prevalence of mental disorders increases throughout adolescence [15], and adolescent participant age at 3-year follow-up would have been 16 years on average, there may have been greater opportunity for parents to engage in mental health first aid with their children, as compared to the earlier 1- and 2-year follow-ups. The primary aims were to evaluate the training’s effect on the support parents offer their adolescent children with a mental health problem and the effect on adolescent mental health. Of secondary interest was whether improvements in mental health literacy were maintained over time.

## Methods

### Study design

Study methods have been described in detail elsewhere [14] but are briefly described here. The study was an RCT with participants randomized to Youth MHFA (YMHFA) or Red Cross Provide First Aid (PFA) training. It was registered with the Australian and New Zealand Clinical Trials Registry (ACTRN12612000390886).

### Participants and randomisation

Participants were Australian parents of an adolescent aged 12–15 years, with one parent and adolescent registered as a dyad. The study was promoted via schools, the media and online. The trial website (www.tpot.net.au) provided information about the study and an online registration and consent form. Randomisation occurred during online registration using a random number generator programmed on the trial website. Two to three weeks after online registration, baseline interviews were conducted by a telephone survey company. These were conducted with both the parent and adolescent independently. Parents were informed of their randomisation at the end of the baseline interview. Blinding of parent participants to intervention was not possible, but telephone interviewers were blinded at each assessment. Recruitment occurred between October 2011 and March 2016.

### Interventions

Accredited YMHFA or PFA instructors delivered the two face-to-face training courses to groups of parents in the community. Adolescents did not receive any training. Participants also received a course manual [16, 17].

#### Youth Mental Health First Aid course

The YMHFA training course is delivered to adults who work with or care for adolescents. The course teaches information about adolescent development, the signs and symptoms of the common and disabling mental health problems in young people, where and how to get help when a young person is developing a mental illness, what sort of help has been shown by research to be effective, and how to provide first aid in crisis situations. The course was taught across 4 × 3.5 h sessions, usually run over two consecutive days.

#### Red Cross Provide First Aid course

The PFA training (HLTAID003) offers the skills and knowledge required to provide first aid response, life support, and casualty management until the arrival of medical or other assistance. Topics covered include cardiopulmonary resuscitation, drowning, anaphylaxis, airway obstruction, bleeding and wound care, head, neck and spinal injuries, poisoning, envenomation, seizure management, stroke and unconsciousness. The 15-h course was taught over 2 sessions.

### Outcomes

Outcomes were assessed via computerized telephone interview at baseline and 1, 2, and 3-years later.

### Primary outcomes

#### Adolescent mental health problem

Cases of a probable mental health problem were assessed by the 25-item Strengths and Difficulties Questionnaire (SDQ) [18] Parent and Child Report versions, adapted for phone interview. Total difficulties scores in the abnormal range (> = 17 for parent report and > =20 for adolescent self-report) indicated the presence of a probable mental health problem [19, 20].

#### Perceptions of parent support by adolescents with a mental health problem

This was assessed by asking adolescents the following questions: (1) “Over the last 12 months, have you had any sort of mental health problem?” If yes, (2) “What do you think the problem was?” (3) “Did you get help from family?”, and (4) “How well did your mother/father support you when you had your mental health problem?” (asked about each adolescent’s parent, as appropriate). Responses were “Very well”, “Fairly well”, “Not well”, or “Unsure”. If the adolescent was unsure what was meant by a “mental health problem”, they were told “a period of weeks or more when you are feeling depressed, anxious, emotionally stressed, or are misusing alcohol or drugs, and these problems are interfering with your life”. Responses were dichotomized into (1) “Very well” versus (0) “Fairly well”, “Not well”, or “Unsure”.

#### Quality of parental support towards adolescents with a mental health problem

This was assessed by questions to the parent adapted from a previous survey [21]. (1) “Over the last 12 months has [your child] had any sort of mental health problem?” If yes, (2) “What do you think the problem was?”, (3) “Over the last 12 months, have you done anything to help him/her with this mental health problem?”, (4) “What did you do?”. Open-ended responses to actions taken were scored blinded to group according to congruence with the MHFA Action Plan. Responses received a score of 0–2 points for each component of the plan, with total scores ranging from 0 to 12 [22].

### Secondary outcomes

The trial included a variety of secondary outcomes addressing mental health literacy, in common with other evaluations of MHFA [7] (see Table 1 for a description of each outcome measure). For parents, these included recognition of mental health problems, knowledge about youth mental health problems, confidence and intentions to help a person with a mental health problem, stigma towards people with mental health problems, and appropriate help-seeking for mental health problems. Some outcomes were in response to 4 hypothetical vignettes describing an adolescent with a mental health problem: depression, social phobia, an eating disorder not otherwise specified and psychosis [14]. In adolescents, secondary outcomes included perceived general social support from parents, intended and actual help-seeking from parents for a mental health problem, help-seeking from a health professional, and stigmatizing attitudes.

**Table 1:** Overview of secondary outcomes

Outcome	Survey measure	Response	Scoring	Psychometrics
**Outcomes completed by both parents and adolescents**				
Social Distance [23]	5 questions assessing desire for social distance from the person in the psychosis vignette. E.g. *Would you be happy for your child / Would you be happy … to develop a close friendship with X?*	4-point Likert scale 1 = Yes definitely 2 = Yes, probably 3 = Probably not 4 = Definitely not	Mean score of 5 questions, range 1–4.	Parent ω = .90 Adolescent ω = .88
Stigma: Weak-not-sick [23]	4 questions assessing that the person in the psychosis vignette is weak not sick. E.g. *X could snap out of it if (he/she) wanted*	5-point Likert scale 1 = Strongly disagree 2 = Disagree 3 = Neither agree nor disagree 4 = Agree 5 = Strongly agree	Mean score of 4 questions, range 1–5, dichotomized to low stigma (=1) or not (> 1).	Parent ω = .81 Adolescent ω = .56
Stigma: Dangerous/Unpredictable [23]	4 questions* assessing that the person in the psychosis vignette is dangerous or unpredictable. E.g. *X’s problem makes (him/her) unpredictable*	5-point Likert scale 1 = Strongly disagree 2 = Disagree 3 = Neither agree nor disagree 4 = Agree 5 = Strongly agree	Mean score of 4 questions, range 1–5.	Parent ω = .46 Adolescent ω = .45
K6 psychological distress (12-month version) [24]	6 questions assessing the frequency of psychological distress in the worst month during the past 12 months	5-point Likert scale 1 = None of the time 2 = A little of the time 3 = Some of the time 4 = Most of the time 5 = All of the time	Total scores of 19 or above indicate a high level of psychological distress	Parent ω = .91 Adolescent ω = .93
**Outcomes completed by parents only**				
Problem recognition	*What, if anything, do you think is wrong with X?* Question in response to each of 4 vignettes: depression, social phobia, EDNOS, psychosis	Verbatim responses were scored for correct/incorrect, blinded to group allocation and timepoint.	1 point for correct recognition if the following were mentioned: Depression (depression, depressed), social phobia (anxiety/anxious, social anxiety, social phobia, anxiety disorder, performance anxiety), EDNOS (Eating disorder, EDNOS, bulimia/bulimic/bulimia nervosa/mia, binge eating/loss of control eating, binge eating disorder, anorexia/anorexia nervosa/anorexic/ana, disordered eating), psychosis (schizophrenia/paranoid schizophrenia, psychosis/psychotic, schizophrenic, schizoaffective disorder, precursor to schizophrenia). Mean score of 4 responses, range 0–1.	ω = 0.82
Quality of MHFA intentions	*Imagine X is your child. You want to help (him/her). What would you do?* Question in response to each of 4 vignettes: depression, social phobia, EDNOS, psychosis	Verbatim responses were scored for consistency with the MHFA Action Plan in the Youth MHFA manual, [17] blinded to group allocation.	Mean score of 4 responses, range 0–12.	Inter-rater reliability (ICC) for 4 vignettes ranged from 0.74–0.84. ω = 0.56
Confidence to help	*How confident would you be in your ability to help X?* Question in response to each of 4 vignettes: depression, social phobia, EDNOS, psychosis	4-point Likert scale 1 = Very confident 2 = Fairly confident 3 = Slightly confident 4 = Not confident at all	Mean score of 4 responses, range 1–4.	ω = 0.84
Knowledge about youth mental health problems	18 questions on knowledge about mental health problems, derived from the Youth MHFA manual [17]. E.g. *If a teenager has a traumatic experience, it is best to make them talk about it as soon as possible*	Agree, Disagree, Don’t know	1 point for a correct response to each question, range 0–18.	Test-retest *r* = 0.67 (PFA group)
Quality of MHFA support towards other person	*Over the last 12 months, has anyone else you know had any sort of mental health problem?...Have you done anything to help the person with this problem?...What did you do?*	Verbatim responses were scored for consistency with the MHFA Action Plan, blinded to group allocation.	Scores range 0–12.	Inter-rater reliability ICC = 0.76
Appropriate help-seeking for mental health problem	*Over the last 12 months, have you yourself had any sort of mental health problem?...Have you done anything to deal with this mental health problem?...What did you do?*	Verbatim responses were scored for the presence of an appropriate source of help.	Scored a 1 if mentioned any of the following professionals or treatments: taking medication, antidepressant, anxiolytic, counselling, counsellor, GP, psychologist, psychiatrist, CBT, hospital, other medical (mental health practitioner or specialist, professional for alcohol misuse, maternal & child health nurse), professional organization (Centre Against Sexual Assault, Drummond Family Services).	
**Outcomes completed by adolescents only**				
Perceived general social support from parent [25]	Communication subscale of Parent Attachment from the People in My Life Questionnaire (5 questions). E.g. *My parents can tell when I am upset about something.*	4-point Likert scale 1 = Almost never or never true 2 = Sometimes true 3 = Often true 4 = Almost always or always true	Scores range 5–20.	ω = 0.82
Intended help-seeking from parent	*If you had a problem right now like X would you ask for help?...Where would you go?* Question in response to each of 4 vignettes: depression, social phobia, EDNOS, psychosis	Verbatim responses.	Scored 1 point per vignette if mentioned seeking help from a parent. Sum score of 4 responses, range 0–4.	ω = 0.71
Actual help-seeking from parent	*Over the last 12 months, have you had any sort of mental health problem?... Did you get help from family?... Who in the family?*	Verbatim responses.	Scored a 1 if adolescent reported seeking help from either or both parents.	
Help-seeking from a health professional	*Over the last 12 months, have you had any sort of mental health problem?... Did you get help from a health professional or counsellor?*	Verbatim responses.	Scored a 1 if adolescent responded with yes.	

Note: ω was calculated as Revelle’s omega total for total scores

*3 questions only for adolescents, see Yap et al. 2014 [23]

### Sample size estimation

As reported in Morgan et al. [14], we required a sample size of 990 parent-adolescent dyads (495 per group). Based on 12-month prevalence data [26, 27], this sample size would include 128 adolescents with a mental health problem (64 in each group). This would allow us to detect an effect size of d = 0.44, with a power = 0.80 and an alpha = 0.05, in the sub-sample of adolescents with a mental health problem.

### Adverse events

There were no harms reported in this trial. Phone numbers for several services offering free 24-h telephone crisis or counselling support were provided to participants in case they felt distressed during the training or phone interviews and they were encouraged to advise the trial manager if they used these resources. A resource on suicide prevention was offered to adolescents aged 18 or older who were asked questions about suicide as part of the telephone interview.

### Ethics

The study was approved by the University of Melbourne Human Research Ethics Committee.

### Statistical analysis

Data were analyzed with mixed-effects models for continuous and binary outcome variables, with group by measurement occasion interactions. To help meet the missing at random assumption, parent age, tertiary education, and speaks only English at home were included as fixed effects in models analyzing parent outcomes. For continuous outcome measures with no substantial baseline imbalance, effect sizes (Cohen’s d) were calculated by dividing the difference between the two group means at follow-up by their pooled standard deviation. With baseline imbalances, Cohen’s d was calculated by dividing the mean change in each condition by the pooled standard deviation at follow-up. Effects were interpreted as small = 0.2, medium = 0.5 and large = 0.8. Analyses were performed in Stata 15 and RStudio and the significance level was set at *p* < .05.

## Results

### Participant flow and numbers analyzed

**Fig. 1:**
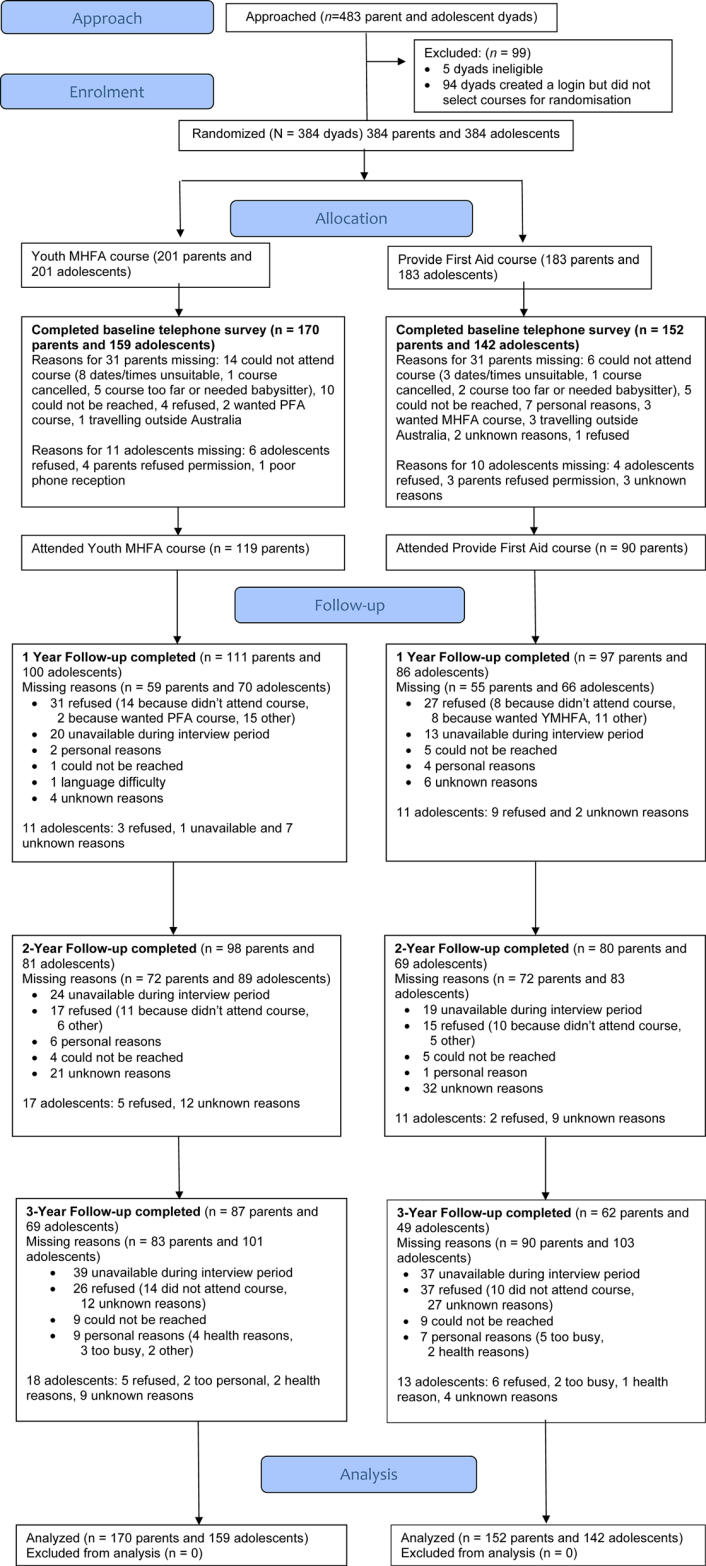
CONSORT flow diagram

The flow of participants throughout the trial is shown in Fig. 1. At 3-year follow-up there were 149 parents with interview data (46.3% of baseline) and 118 adolescents (36.6%). Logistic regression models explored predictors of missingness at 3-year follow-up. Parents were less likely to be missing if they had a tertiary education (OR = 0.58, 95% CI 0.33 to 0.95), were younger in age (OR = 0.95, 95% CI 0.90 to 0.99) and spoke only English at home (OR = 0.38, 95% CI 0.19 to 0.76).

### Participant characteristics

At baseline the two intervention groups showed similar sociodemographic characteristics (see Table 2). At the 3-year follow-up, adolescents had a mean age of 16.5 (SD = 1.15) and slightly more than half were female (*n* = 82, 55%). Only one parent had attended a MHFA course in the previous 12 months (in the MHFA group) and training in other mental health courses was rare (3.2% of PFA group, 6.9% of MHFA group).

**Table 2:** Baseline participant characteristics

	YMHFA (***n*** = 170)	PFA (***n*** = 152)	TOTAL (***n*** = 322)
**Parent**			
Family situation %			
Child living with both parents	71.8	75.7	73.6
Parents separated but both involved in care of child	12.9	9.2	11.2
Parents separated but only respondent involved in care of child	7.1	5.3	6.2
Sole parent	6.5	6.6	6.5
Other (e.g. grandparents)	1.8	3.3	2.5
Married/defacto %	74.1	80.9	77.3
Female %	89.4	86.8	88.2
Tertiary education %	56.6	50.7	53.8
Speaks only English at home %	85.9	79.6	82.9
Not Aboriginal or Torres Strait Islander %	99.4	98.7	99.1
Employed full or part time %	66.5	71.7	68.9
Studying status %			
Full-time	2.9	3.9	3.4
Part-time	16.5	11.2	14.0
Not studying	11.2	18.4	14.6
Unknown	69.4	66.4	68.0
Age M (SD)	45.2 (5.54)	45.1 (5.69)	45.2 (5.60)
Number of children M (SD)	2.2 (0.8)	2.3 (1.0)	2.2 (0.9)
**Child**			
Female %	58.8	52.0	55.6
Health issue %	26.5	27.0	26.7
Age M (SD)	13.3 (1.11)	13.3 (1.08)	13.3 (1.09)

### Primary outcomes

Table 3 shows the primary outcomes at baseline and 3-year follow-up for the physical first aid and Youth Mental Health First Aid groups. There was a smaller proportion of adolescents with a mental health problem in the MHFA group than the PFA group at 3-year follow-up, based on both parent and child report on the SDQ, but the difference over time was not statistically significant.

**Table 3:** Primary outcomes at baseline and 3-year follow-up for Physical First Aid and Youth Mental Health First Aid groups

																	Change over time between YMHFA and PFA
	**Baseline**				**1-year follow-up**				**2-year follow-up**				**3-year follow-up**				**Baseline to 3-year follow-up**
	**PFA**		**YMHFA**		**PFA**		**YMHFA**		**PFA**		**YMHFA**		**PFA**		**YMHFA**		
	**n/N**	**%**	**n/N**	**%**	**n/N**	**%**	**n/N**	**%**	**n/N**	**%**	**n/N**	**%**	**n/N**	**%**	**n/N**	**%**	**OR (95% CI)**
Adolescent with mental health problem – parent reported^a^	19/151	12.6	31/168	18.5	8/96	8.33	15/109	13.8	10/80	12.5	14/98	14.3	6/61	9.8	6/86	7.0	0.17 (0.01 to 1.98)
Adolescent with mental health problem – adolescent reported^b^	11/142	7.75	20/159	12.6	6/86	6.98	12/100	12.0	5/69	7.25	5/81	6.17	5/49	10.2	4/69	5.8	0.16 (0.02 to 1.41)
Mother supported adolescent very well^c^	14/26	53.9	17/30	56.7	6/10	60.0	8/22	36.4	2/11	18.2	8/20	40.0	4/15	26.7	10/23	43.5	2.80 (0.11 to 72.56)
Father supported adolescent very well^c^	7/23	30.4	7/29	24.1	3/8	37.5	2/21	9.52	2/10	20.0	5/19	26.3	3/15	20.0	8/24	33.3	4.31 (0.31 to 60.68)
	**PFA (*n*** **= 38)**		**MHFA (*n*** **= 54)**		**PFA (*n*** **= 24)**		**MHFA (*n*** **= 28)**		**PFA (*n*** **= 18)**		**MHFA (*n*** **= 34)**		**PFA (*n*** **= 19)**		**MHFA (*n*** **= 35)**		**Baseline to 3-year follow-up**
	**M**	**SD**	**M**	**SD**	**M**	**SD**	**M**	**SD**	**M**	**SD**	**M**	**SD**	**M**	**SD**	**M**	**SD**	**M**_**diff**_ **(95% CI), d (95% CI)**
Quality of parental support towards adolescents with mental health problem	2.55	1.18	2.41	1.19	2.29	1.08	2.46	1.00	2.61	1.09	2.82	1.45	2.32	0.95	2.77	1.33	0.41 (−0.38 to 1.20), 0.38 (− 0.18 to 0.94)

^a^Identified by total difficulties scores in the abnormal range of 17 or greater on the Strengths and Difficulties Questionnaire Parent Report

^b^Identified by total difficulties scores in the abnormal range of 20 or greater on the Strengths and Difficulties Questionnaire Child Report

^c^Versus “Fairly well”, “Not well”, or “Unsure” combined (reference category)

*M* Mean, *SD* Standard deviation

Parents who reported their adolescent had a mental health problem generally scored low on the quality of mental health first aid support given to their child. Across follow-up, scores were higher in the MHFA group relative to the control, and although the MHFA group showed a small-to-medium sized improvement (d = 0.38) at 3-year follow-up, this change was not statistically significant. Unfortunately, parents gave truncated responses to the question of what they did to help their adolescent. Responses were only 16–17 words long on average with only superficial detail provided.

Perceptions of support from parents by adolescents with a mental health problem were more positive in the MHFA group. However, the odds of improving between baseline and 3-year follow-up, relative to the control group, were not significant.

### Secondary outcomes

The MHFA group showed small-to-medium improvements at 3-year follow-up relative to the control in two secondary outcomes: parental knowledge about youth mental health problems and adolescent perceptions of general social support from their parents (see Table 4). Of note, adolescent self-reported help-seeking for a mental health problem from their parent or a health professional was higher in the MHFA group than the PFA group, but the number of adolescents reporting a problem was very small (15 in PFA and 26 in MHFA) so the difference was not significant. Most other outcomes showed small or very small effects, which were not significant.

**Table 4:** Secondary outcomes at baseline and 3-year follow-up for Physical First Aid and Youth Mental Health First Aid groups

	Baseline						3-year follow-up						Change over time between MHFA and PFA
	PFA			MHFA			PFA			MHFA			Baseline to 3-year follow-up
**Variable**	**n**	**M**	**SD**	**n**	**M**	**SD**	**n**	**M**	**SD**	**n**	**M**	**SD**	**M**_**diff**_ **(95% CI),** **d**^**#**^ **(95% CI)**
MHFA knowledge	152	9.77	2.76	170	10.28	2.71	62	9.95	2.62	86	12.00	2.13	0.96 (0.26 to 1.66)^**^, 0.31 (−0.02 to 0.63)
Problem recognition	152	0.63	0.35	170	0.66	0.31	62	0.76	0.28	86	0.86	0.23	0.03 (−0.05 to 0.11), 0.07 (− 0.25 to 0.40)
Quality of MHFA intentions	152	2.24	0.62	170	2.34	0.73	62	2.29	0.78	86	2.58	0.84	0.12 (−0.15 to 0.39), 0.09 (− 0.23 to 0.42)
Confidence	151	2.25	0.68	169	2.36	0.61	62	2.13	0.72	86	2.08	0.59	−0.11 (−0.29 to 0.08), 0.01 (− 0.32 to 0.33)
Quality of MHFA support towards other person	86	2.23	1.16	113	2.29	1.14	34	2.18	1.34	58	2.47	1.14	0.21 (−0.35 to 0.76), 0.24 (− 0.19 to 0.66)
Social distance (parent)	145	2.16	0.66	160	2.08	0.63	61	1.89	0.67	85	1.79	0.58	0.05 (−0.12 to 0.21), − 0.05 (− 0.38 to 0.29)
Dangerous/ unpredictable stigma (parent)	147	2.48	0.52	164	2.44	0.56	61	2.31	0.60	85	2.26	0.56	0.03 (−0.16 to 0.21), − 0.09 (− 0.41 to 0.24)
Social distance (adolescent)	140	2.23	0.61	157	2.20	0.66	47	2.22	0.64	69	2.18	0.60	−0.06 (−0.25 to 0.13), 0.10 (− 0.28 to 0.47)
Weak not sick stigma (adolescent)	142	2.33	0.65	157	2.29	0.75	48	1.62	0.58	69	1.54	0.63	0.08 (−0.15 to 0.30), − 0.24 (− 0.61 to 0.13)
Dangerous/ unpredictable stigma (adolescent)	133	2.63	0.67	148	2.56	0.67	48	2.56	0.52	65	2.46	0.67	−0.08 (−0.31 to 0.14), 0.19 (− 0.20 to 0.57)
Perceived general social support from parent	142	16.26	3.11	159	15.74	3.50	49	14.37	3.30	69	15.48	3.28	1.15 (0.09 to 2.21)^*^, 0.35 (−0.02 to 0.72)
Adolescent intended help-seeking from parent	140	2.71	1.18	158	2.38	1.27	48	2.69	1.36	69	2.46	1.43	0.03 (−0.43 to 0.48), 0.03 (− 0.34 to 0.40)
	**n**	**%**		**n**	**%**		**n**	**%**		**n**	**%**		**OR (95% CI)**
Weak not sick stigma (parent) – median or below	87	57.6		95	56.6		46	74.2		70	81.4		1.06 (0.29 to 3.83)
K6 above cut-off (parent)	28	18.5		37	21.9		12	19.7		13	15.1		0.38 (0.09 to 1.69)
K6 above cut-off (adolescent)	33	23.4		42	26.6		16	33.3		21	30.4		0.71 (0.17 to 3.02)
Parent sought appropriate help for own mental health problem	36	81.8		49	69.0		12	75.0		24	75.0		10.24 (0.27 to 384.71)
Adolescent sought help from parent for mental health problem	22	75.9		24	77.4		6	40.0		17	65.4		4.69 (0.36 to 61.64)
Adolescent sought help from health professional for mental health problem	19	65.5		23	74.2		9	60.0		19	73.1		1.16 (0.15 to 8.87)

*p < .05, **p < .01

# Positive Cohen’s d favors MHFA and negative d favors PFA

*M* Mean, *SD* Standard deviation

## Discussion

This paper reports on the 3-year outcomes from Youth MHFA training as part of the Training for Parents of Teenagers randomized controlled trial. Overall, results at 3-year follow-up were similar to those observed at 1- and 2-year follow-up [14]. Notwithstanding the limitations imposed by under-recruitment and high levels of attrition, the trial could not demonstrate clear benefits to the recipients of mental health first aid. Although effects were in the direction favoring MHFA training, the number of participants at follow-up was low, and the sub-sample reporting a mental health problem in adolescents even smaller. As with earlier follow-ups, there remained a lack of statistical power at 3-year follow-up. Even though there were higher rates of adolescents with mental health problems at 3-year follow-up (reflecting the higher risk period of middle-to-late adolescence), there was more attrition compared to 1- and 2-year follow-up [14]. There were few improvements in study outcomes observable 3 years after training, apart from parents’ knowledge of youth mental health problems and adolescents’ perceptions of general social support from their parents. Improved parent mental health knowledge was the largest effect demonstrated at 1- and 2-year follow-up, and this was sustained at 3-year follow-up. The effect on adolescents’ perceptions of general support from parents was stronger at 3 years compared to earlier timepoints, but it is possible this is a chance finding, given the large number of outcomes analyzed. Most other mental health literacy effects had faded and were too small to detect with our sample size.

The results from this study support the need for refresher MHFA training to maintain knowledge and skills. Refresher courses in Youth, Standard (adult) and Aboriginal and Torres Strait Islander MHFA are now offered by Mental Health First Aid Australia. These are 4-h face-to-face courses for those who have completed training in the last 3 years. The Refresher Courses are designed to update knowledge and skills and include a focus on attendees’ experiences providing mental health first aid, barriers and facilitators to providing aid, and knowing when to approach someone. In line with physical first aid accreditation, completion of refresher courses can extend MHFA accreditation for a further 3 years. Refresher Courses have not yet been evaluated, but Mental Health First Aid Australia plans to do so as part of its research strategy.

Future research should investigate other ways of testing whether MHFA training benefits the recipient of aid, in designs where the trainee and aid recipient can be participants in the study. One approach is to train employees in an organization, who can serve as both trainees and potential recipients of aid from co-workers. The EMPOWER study is currently being conducted by the Centre for Mental Health and London South Bank University in the United Kingdom. This study will provide MHFA training to selected staff in workplaces and examine employee experiences of providing and receiving aid, as well as effects on mental health and economic impacts [28]. Although mental health first aid provided within a workplace setting may not generalize to other settings or relationships, a workplace setting can provide objective data on sickness rates and productivity and allows for an examination of changes in organizational culture. Another way to evaluate the effect of MHFA training on aid recipients includes training school staff in Youth MHFA and examining the impact on student help-seeking and mental health. This design allows for the collection of objective data on referral rates to school counselling services, which avoids relying on student self-reports of help received from others, which was assessed in a previous school-based trial [29]. A similar approach would be to train young people in university or college settings, who could then provide support to college peers, and data on help-seeking could be obtained from college counselling services (e.g. [30]).

Another issue for future research on MHFA is how to capture quality data on changes in mental health first aid skills. This study used open-ended responses to collect data on intentions to give aid and descriptions of aid provided. These responses were then scored against the MHFA Action Plan. As reported above, most of the descriptions given by parent participants were very brief and superficial in detail and therefore corresponded to low scores. The experience from this trial and others (e.g. [31, 32]) shows that this method may not capture the full extent of the action participants took, making it difficult to show changes over time. An alternative is to present a list of potential actions for participants to select from, including actions consistent with MHFA training and plausible but inconsistent actions, such as has been used in suicide prevention research [33]. This approach would mean all aspects of the MHFA Action Plan could be assessed and it would avoid the problem of truncated and superficial responses. Repeated brief assessments at shorter intervals than 1-year follow-up would also minimize the impact of memory on recollecting supportive or helpful behaviours offered.

This study’s key strengths were its long follow-up period to assess the lasting impact of any training effects, the active control condition to control for receiving health education training, and involvement of both parents and adolescents to assess training effects upon aid recipients. Notwithstanding these strengths, the study had some significant limitations. There was a high degree of attrition over time, which limited power to detect training effects. Attrition at follow-up was partly due to a lack of interest in the study from parents who did not attend their allocated course. These were a significant minority and more frequent in the control group. Furthermore, using an independent survey company to contact participants at follow-up may have reduced the potential for successful cohort maintenance by the research team. Finally, one of our primary outcome measures, cases of adolescent mental health problems, may have been too far downstream to be a realistic outcome to measure. MHFA is not designed to prevent or treat mental health problems, and improvements in the mental health of aid recipients depends on the quality of professional help received. Focusing on intermediary steps, such as rates of professional help-seeking, may be a more suitable primary outcome measure to assess in future trials of MHFA.

## Conclusions

Three years after training, participants had maintained their improved knowledge about youth mental health problems. There were some indications of positive effects upon adolescent mental health and parental support given to adolescents with a mental health problem, but the study was underpowered to clearly show benefits to mental health first aid skills and recipients of aid.

## Data Availability

Requests for access to the data should be made to the first author.

## Abbreviations

EDNOS: Eating Disorder Not Otherwise Specified
MHFA: Mental Health First Aid
OSFED: Other Specified Feeding and Eating Disorder
PFA: Provide First Aid
SDQ: Strengths and Difficulties Questionnaire
YMHFA: Youth Mental Health First Aid
